# Advanced Strategies for the Regeneration of Lumbar Disc Annulus Fibrosus

**DOI:** 10.3390/ijms21144889

**Published:** 2020-07-10

**Authors:** Javad Tavakoli, Ashish D. Diwan, Joanne L. Tipper

**Affiliations:** 1 Centre for Health Technologies, School of Biomedical Engineering, Faculty of Engineering and Information Technology, University of Technology Sydney, Ultimo 2007, Australia; A.Diwan@spine-service.org; 2 SpineLabs, St George and Sutherland Clinical School, The University of New South Wales, Sydney 2052, Australia; 3 Spine Service, Department of Orthopaedic Surgery, St George Hospital Campus, Kogarah 2217, Australia

**Keywords:** intervertebral disc, annulus fibrosus, repair, regeneration, herniation, cell-therapy, gene-therapy, biomolecule-therapy

## Abstract

Damage to the annulus fibrosus (AF), the outer region of the intervertebral disc (IVD), results in an undesirable condition that may accelerate IVD degeneration causing low back pain. Despite intense research interest, attempts to regenerate the IVD have failed so far and no effective strategy has translated into a successful clinical outcome. Of particular significance, the failure of strategies to repair the AF has been a major drawback in the regeneration of IVD and nucleus replacement. It is unlikely to secure regenerative mediators (cells, genes, and biomolecules) and artificial nucleus materials after injection with an unsealed AF, as IVD is exposed to significant load and large deformation during daily activities. The AF defects strongly change the mechanical properties of the IVD and activate catabolic routes that are responsible for accelerating IVD degeneration. Therefore, there is a strong need to develop effective therapeutic strategies to prevent or reconstruct AF damage to support operational IVD regenerative strategies and nucleus replacement. By the way of this review, repair and regenerative strategies for AF reconstruction, their current status, challenges ahead, and future outlooks were discussed.

## 1. Introduction

**Figure 1:**
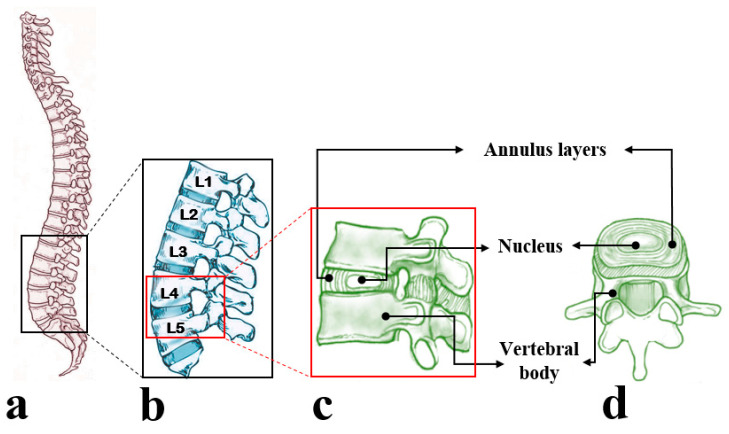
Schematic presentation of (**a**) the human spine and (**b**) lumbar region of the human spine consisting of five intervertebral discs (IVDs). (**c**,**d**) Represent a functional spinal unit including IVD and adjacent vertebral bodies from the lumbar spine displayed from the side and top views, respectively. The vertebral body, nucleus, and annulus layers in an isolated IVD were identified using black arrows. The lumbar spine and a functional spinal unit (side view) under two magnifications were denoted by black and red rectangles, respectively.

The intervertebral disc (IVD), located between adjacent vertebral bodies in the spine, undergoes large deformations and bears significant loads during daily activities. By occupying approximately one-third of the spinal column’s height, IVDs play a significant role in spinal motion and provide flexibility to the spinal column [1]. The IVD consists of thick outer fibrocartilaginous rings called the annulus fibrosus (AF) that surrounds an inner gelatinous core known as the nucleus pulposus (NP) (Figure 1). 

Degeneration of IVD is a complex process and its association with low back pain (LBP) is not very well known; however, studies have shown aging, genetic, occupation, and IVD disruption may lead to IVD degeneration causing LBP [2,3,4,5]. LBP is a prevalent and debilitating condition with extensive socio-economic impacts affecting the quality of life, reflected by the years lived with disability (YLD). YLD is a measure of the burden of diseases indicating the prevalence of a disorder by loss of health associated with that disability. LBP is the foremost reason for YLD and the second most common cause of hospital visits with the associated direct expenditure of $1.2 billion in Australia, exceeding $100 billion in the US annually [6,7,8]. 

**Figure 2:**
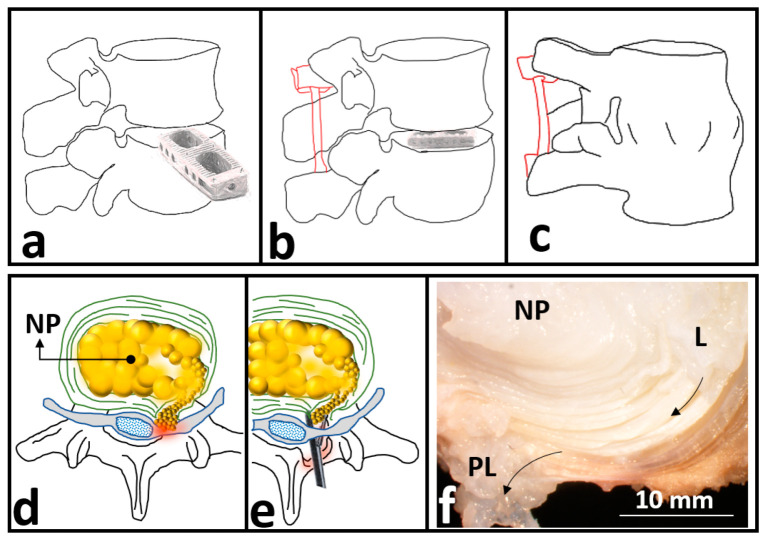
(**a**–**c**) Schematic drawing for spinal fusion to treat degeneration-induced-low back pain (LBP) including (**a**) remove the degenerated intervertebral disc (IVD) (**b**) insertion of spin polyetheretherketone (PEEK) cage and (**c**) restrain the adjacent vertebrae for bone formation. The schematic drawings in red (**b**,**c**) denoting an internal fixator used in spinal fusion surgery. (**d**,**e**) Schematic drawing of discectomy to treat herniation-induced-LBP including (**d**) a herniated IVD and (**e**) the percutaneous endoscopic discectomy process to alleviate LBP by removing the herniated nucleus polposus (NP). (**f**) A camera photo captured from a herniated IVD to identify herniation pathway initiating from the lateral region (L) moving towards the posterolateral region (PL) of the AF donated by black arrows. (Panel f was reproduced with permission from [18]).

Degenerative changes in spinal IVDs are frequently detected in patients suffering LBP. Current treatments for degeneration-induced-LBP include conservative methods (i.e., rehabilitation) and surgical interventions depending on the severity of IVD degeneration [9,10]. To reduce LBP in highly degenerative IVDs, the current gold standard surgical treatment is spinal fusion aiming to remove the degenerated IVD to finally restrain the adjacent vertebrae (Figure 2a–c). Unfortunately, by fusing the adjacent vertebrae, the method limits the spine mobility and accelerates the degeneration of the adjacent IVDs [11]. Another surgical intervention to replace NP or total IVD, arthroplasty, maintains the spine motion; however, suffers long recovery with notable post-surgery complications. Total IVD implants may induce bone problems in the long term and are not appropriate for patients suffering from osteoporosis or osteopenia [12]. Moreover, the generation of volumetric wear and particulate debris which is likely to occur after total IVD replacement is a concern [13]. While the biological response to the size and volume concentration of generated particles is known to play a key role in osteolysis in other artificial joints, more studies are required to address the concern in IVD implants [14,15,16].

Apart from degeneration, IVD structural defects and damages to the AF are likely to result in herniation causing LBP [17]. Herniation occurs when the NP soft material leaks through the AF of the IVD. Discectomy is the current gold standard surgical intervention to treat herniation-induced-LBP (Figure 2d–f), which aims to remove the herniated NP materials. Consequently, discectomy involves removing a portion of the AF leading to an undesirable condition that may accelerate IVD degeneration causing chronic LBP.

Unfortunately, surgical interventions suffer limitations, and their failure to induce regeneration or stop IVD degeneration is the main drawback. To overcome the inherent restrictions associated with surgical methods to cure LBP, recent investigations have been focused on IVD regenerative medicine with growing interests in cell, biomolecules, and gene therapies [19,20,21,22,23]. Despite intense research interest, attempts to regenerate the IVD have failed so far and no effective strategy has translated into a successful clinical outcome [24,25]. Of particular significance, the failure of strategies to repair the AF has been a major drawback in the regeneration of IVD and nucleus replacement [26]. Since IVD is exposed to significant load and large deformation during daily activities, it is unlikely to secure regenerative mediators (cells, genes, and biomolecules) and artificial nucleus materials after injection into a herniated IVD. 

Since AF repair after herniation is still an unsolved challenge, the current review paper aimed to present studies on the repair and regeneration of the AF. This systematic review was undertaken by a search through Science Direct and PubMed (NLM) online databases published in peer-reviewed journals. The search keywords were “annulus fibrosus” AND/OR “repair”, AND/OR “regeneration” covering the period from 2015 to 2020. The bibliographies of these papers were also used to identify additional relevant papers that did not appear in the keyword search.

## 2. The Anatomy and Physiology of the Annulus Fibrosus

### 2.1. Structure and Composition of the Annulus Fibrosus

**Figure 3:**
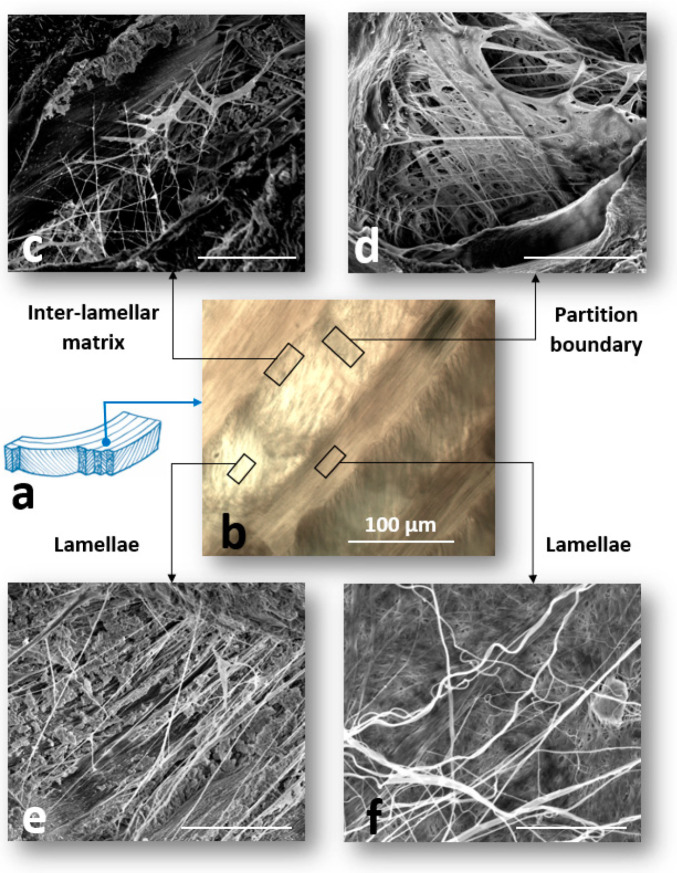
(**a**) Schematic drawing of the annulus fibrosus (AF) lamellar structure. (**b**) A light microscopic image of the AF prepared at a transverse cutting plane. (**c**–**e**) Scanning electron microscopy (SEM) images of the structural organization of elastic fibers in different regions of the annulus fibrosus (AF) including (**c**) inter-lamellar region (ILM), (**d**) partition boundary (PB), and (**e**,**f**) adjacent lamellae. The regions of interest including ILM, PB, and adjacent lamellae in the AF were denoted by black rectangles and the scale bars for the SEM images were 5 μm. (Panels (c,e), panel (d) and panel (f) were reproduced with permission from [34], [30], and [31], respectively).

The AF comprises a unique and complex structure of concentric annular lamellae (Figure 3a). Each lamella consists of highly packed collagen fibers (mainly type I) that are oriented at approximately 30° to the transverse plane, varying to −30° relative to the adjacent lamella. The thickness of the lamellae in the AF varies in the radial direction and is approximately 130 µm for the outer AF [27]. The inter-lamellar matrix (ILM), with a thickness of less than 30 µm, located between each adjacent lamellae (Figure 3b). The ILM is composed of elastic fibers, cells, and non-fibrillar matrix consisting of water, lipids, and proteoglycans. A number of glycoproteins including aggrecan, lubricin, GAGs, biglycan, decorine, perlecan, and versican are the main components of the ILM non-fibrillar matrix, which are responsible for lubricating and sustainable hydration of the ILM [28,29]. Additionally, the adjacent ILMs in the AF are connected by partition boundaries (PBs) that provide structural connectivity between the collagen bundles in the AF lamella (Figure 3b) [30].

Recent studies based on the sonication of the AF samples in NaOH solution to remove non-elastin components (collagen, micro-fibrils, and matrix) revealed a loose network of elastic fibers in the AF lamellae [31,32]. The structure of the elastic fiber network in the ILM is considerably more complex compared to the adjacent lamella with a well-organized network of thick and thin elastic fibers forming a network across the ILM [33]. Elastic fibers present a dense network across the ILM orientating at ± 45° and 0° relative to the collagen fibers, creating a highly organized orthotropic network (Figure 3c–f) [34]. PBs consist of a dense structure of elastic fibers, with a lower density compared to the lamella and a higher density compared to those located in the ILM, but similar fiber orientations [30].

### 2.2. Annulus Fibrosus Cells

The notochords, a rod-like cartilaginous skeletal structure derived from the mesoderm, provide a platform for early development of IVDs in the spine [35]. Multistage mechanisms, including biochemical reactions and biomechanical scenarios, are involved to develop mature cells from notochords. The AF cells in a mature IVD are fibrochondrocytes and chondrocytes, which morphologically are similar to the fibroblasts [36]. These cells, with a higher density in the outer region of the AF ≈ 9 × 106 cm^−3^, have thin and elongated morphology, as found in meniscus tissue [37].

Within the ILM region, different cell morphologies have been reported. In the inner lamellae of the AF with less organized collagen fibers, ILM cells are flattened into a disc shape, and become elongated in the outer AF [38]. The difference in the ILM cells’ structure and morphology is due to different mechanical loads experienced by the outer compared to the inner AF. Within the ILM region, the morphology of the cells changes from circular to fusiform from the center towards the ILM-lamella interface [39]. The change in the morphology of the ILM cells is likely to be influenced by the elastic fibers’ orientation and density, as well as loading direction and magnitude experienced by the adjacent lamella [28].

## 3. Repair Strategies for the Annulus Fibrosus

**Figure 4:**
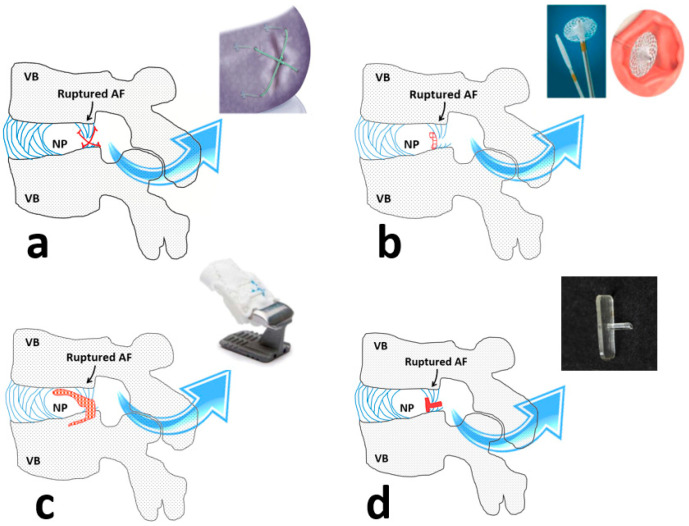
(**a**–**c**) The schematic drawings and images of three different commercial approaches to repair the annulus fibrosus (AF) including (**a**) Xclose^®^, (**b**) Inclose^®^, and (**c**) Barricaid^®^. (**d**) A schematic drawing representing a shape memory polymeric implant that was prepared at the laboratory level to repair the AF. The rupture site at the AF and implant images were denoted by black and blue arrows, respectively. The schematic drawings of the implants were denoted in red sketches for each panel. VB, NP, and AF were abbreviations for the vertebral body, nucleus pulposus, and the annulus fibrosus, respectively. (Panels (c,d images) were reproduced with permission from [53] and [55], respectively).

The main challenge associated with the AF repair is the lack of a functional implant integrating host tissue and seal the site of rupture with the mechanical properties similar to the native counterpart. Previous attempts have failed to address the challenge and successfully repair the AF. Early commercial products to close the rupture site in the AF were Xclose^®^ and Inclose^®^ presented by Anulex Technologies Inc (USA). Xclose ^®^ utilized braided polyester bands to make an X-shaped stitch over the AF defect and Inclose^®^ was a surgical mesh to block the AF rupture and prevent re-herniation (Figure 4a,b). Apparently, these products were discontinued since they have failed to neither withstand high mechanical loads experienced by the AF nor improving the healing rate and strength [40].

In the absence of an effective closure device, suturing techniques have been recruited; however, inconsistent results to address the efficacy of sutures have been reported. In vitro porcine cadaveric studies showed that modified suturing techniques might provide effective closure to withstand 4000 cycles of flexion/extension with 1500  N of axial loading or resist cyclic compressive loading (≈3000 N) [41,42,43]. However, improvement of healing strength was not observed in ovine sutured IVDs [44]. Further biomechanical assessments revealed that closure of the AF by sutures was not reliable to sustain more than 3400 fatigue cycles [45]. 

Minimally invasive thermal procedures including “IVD pulse radiofrequency” and “intra-discal electro-thermal” therapies were likely to repair AF with mild rupture. These techniques provided thermal energy to the AF layers in order to denature collagen fibrils locally and seal the rupture site. Severe AF defects are unlikely to be treated by these methods and their contribution to IVD degeneration in the long term is not known [46,47,48,49,50].

Recently, an AF closure device (Barricaid^®^), with a metallic-base implant and woven polyester component, was introduced (Figure 4c). Barricaid^®^ intended to block the disrupted AF while anchoring to the adjacent vertebral body with a titanium bone anchor [51]. With a successful outcome for reducing pain and decreasing the rate of re-herniation, Barricad^®^ seemed not to maintain or restore native tissue structure and mechanical function [52,53]. This restriction might initiate or accelerate IVD degeneration [54].

Over decades, different polymeric materials for the AF repair have been used in research leading to the creation of several implants and bio-adhesives [56]. The use of fibrin adhesives has improved IVD function and inflammatory response with pain reduction in selected patients suffering from the disco-genic disease [57,58]. A study revealed that the injection of collagen gels crosslinked by riboflavin into the rat IVDs inhibited the progression to the degeneration after needle punctuation [59]. Several studies have shown that the injection of genipin resulted in the integration of AF tissue in degenerated IVD temporarily and improved interfacial shear properties between the AF lamellae [60,61,62,63]. The application of different natural polymers (collagen, alginate, chitosan, silk, and hyaluronan) and synthetic biomaterials (lactic-glycolic composites, poly (1, 8-octanediol malate) and polycaprolactone) for the AF repair have been reported in several studies [64,65,66,67,68,69]. To fabricate collagen-based implants, collagen fibers have been harvested from animal tail tendons and were cross-linked in situ using riboflavin or ammonia [70,71,72,73,74]. A shape memory implant was introduced utilizing macromere of D, L-lactide, and trimethylene carbonate, demonstrating shape recovery at a temperature range of 10 to 40 °C (Figure 4d) [55]. Poly(trimethylene carbonate) combined with an elastic polyurethane membrane was shown to be effective in preventing bovine IVD re-herniation under dynamic loading for 14 days [75]. Amongst studies that suggested polycaprolactone for AF repair, one study introduced a biodegradable construct using poly(caprolactone triol malate) with adjustable degradation and mechanical properties during the pre-polymerization process [76]. Despite successful preliminary laboratory trials to repair AF, the current approaches using polymers lacked appropriate properties (i.e., adhesion) and long-term reliability to resist high stresses during daily activities [77,78,79,80]. Table 1 indicates the current status and the feasibility of the repair strategies that have been used for the AF repair.

## 4. Regenerative Strategies

**Figure 5:**
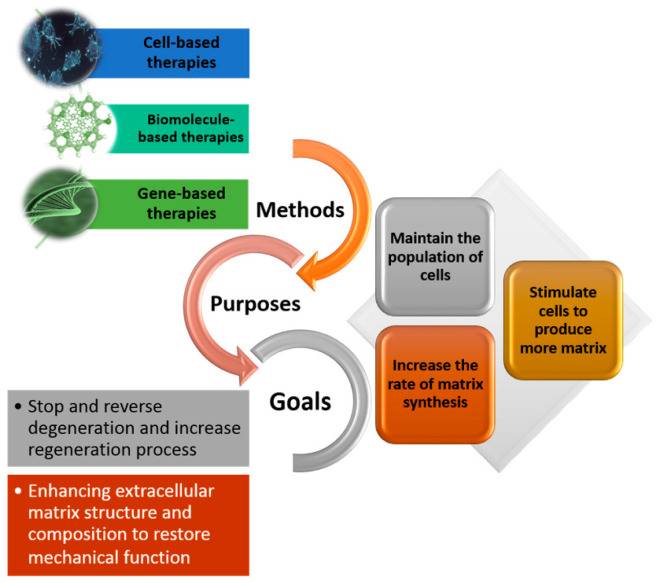
Strategies for the regeneration of the annulus fibrosus: Methods, purposes, and final goals.

Degeneration of the IVD is a complex condition with multifactorial etiology involving age, genetics, and biology. Additionally, structural defects and damages to the AF manifested by mechanical loads are likely to result in IVD herniation and accelerating degeneration. In a degenerated IVD, the AF is less organized and dehydrated with lower proteoglycans exhibiting different mechanical properties compared to the healthy tissues. A decrease in the AF cell density, as frequently observed in degenerated IVDs, alters the balance of anabolism:catabolism ratio in the extracellular matrix (ECM), changing the AF mechanical, biological, and structural properties [69,70,71]. Therefore, regenerative strategies aim to repopulate a degenerated IVD with healthy cells (cell therapy) or stimulating the existing cells to regulate the ECM reaching an enhanced composition, quantity, and structure (biomolecule and gene therapy) [81,82,83,84,85]. ECM remodeling is crucial to control the action of biochemical mediators such as cytokines (interleukins), growth factors, and enzymes (metalloproteinases and heparanase) to ultimately stop or reverse the IVD degeneration process [86,87,88,89]. In the past few years, cell, biomolecule, and gene therapies have been central to the regenerative strategies (Figure 5).

Gene therapies or the addition of specific bone morphogenetic proteins were shown to accelerate the regeneration process, stimulate ECM production, or prevent the progression of annular injuries [90,91,92,93,94]. However, the delivery of genes into the AF cells has been technically challenging due to the low population of cells. Moreover, the selection of appropriate therapeutic genes, while important, is difficult and requires extensive knowledge of the pathogenesis of degradation, specifically timing and stage of degeneration. Besides, the AF cell apoptosis in degenerated IVDs is a barrier to the successful execution of gene-based therapies [95,96,97]. 

The effect of growth differentiation factors (GDFs) to enhance the anabolism-catabolism ratio in the ECM has been considered as an effective biomolecule-based regenerative strategy [93,97]. An increasing body of evidence indicated that GDFs were central to the IVD homeostatic processes, upregulation of the healthy cell marker genes in degenerative cells, and differentiation of mesenchymal stem cells to the NP cells [98,99,100]. Of particular significance, the efficacy of recombinant protein GDF6 has been shown to enhance stem cells’ immobilization leading to healing of the AF and regenerating the NP in ovine and lapin models and improved pain in a rat-allograft model [92,93,101,102]. This paracrine function of GDFs’ may off set the short lifetime in vivo which has been considered a limitation restraining their efficacy to regenerate the AF [103]. Quite likely that the secondary chemotactic action, of GDF6, led to the reported repair of the annular defect [98]. However, GDF’s efficacy to enhance the structural integrity of the AF in a short time (weeks) leading to biomechanical stability of the AF needs more investigations.

**Figure 6:**
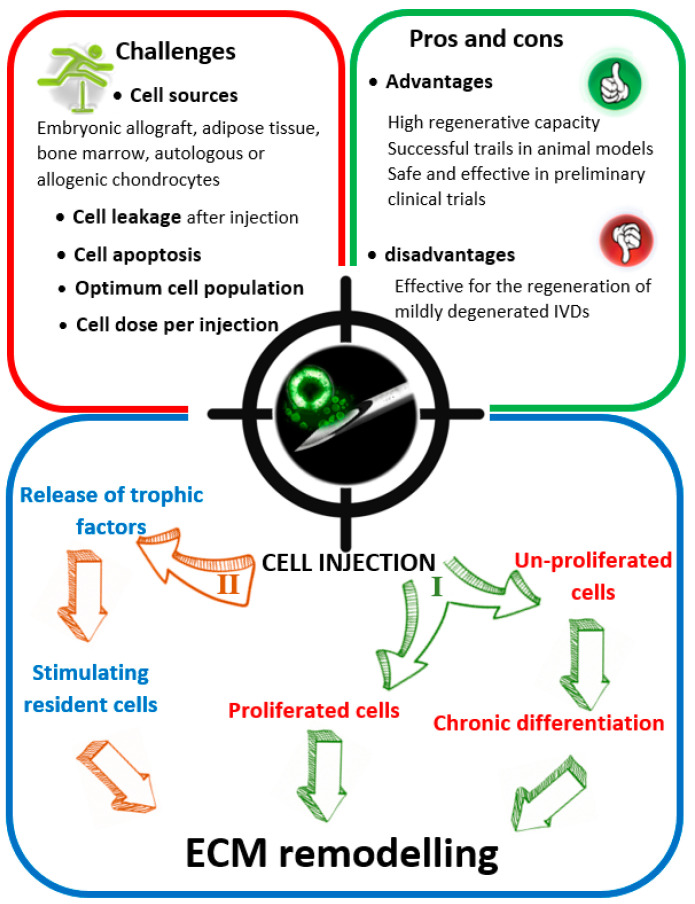
Cell-based regenerative strategy seems the most reliable method for annulus fibrosus (AF) regeneration in mildly degeneratedintervertebral discs (IVDs). Two mechanisms are believed to regenerate the AF effectively. The first mechanism (**I**) is a direct pathway, where the proliferated (or un-proliferated) stem cells contribute to producing extracellular matrix and collagen. The second mechanism (**II**) involves the injection of the stem cells to encourage resident components (mainly cells) to produce extracellular matrix, indirectly.

Challenges associated with genetic modification of resident cells through gene transfer or mobilizing endogenous progenitor cells at the degenerated site, making cell therapy more feasible. In addition, the isolation and culture of mesenchymal stem cells have been well documented [102,104,105]. Different external cell sources, including bone marrow stem cells and mesenchymal stromal cells, have been used for AF regeneration [106,107]. Additionally, proliferated mesenchymal stem cells after direct transplantation into the degenerated AF have shown to play a role in the ECM remodeling to enhance the regeneration process (Figure 6).

However, there are some limitations relevant to cell-based therapy to regenerate the AF [108]. The optimal cell source (bone marrow stem, mesenchymal stromal, and IVD cells) for clinical applications is yet to be defined [106,107]. Preclinical studies have revealed that cell therapy was unlikely to regenerate the AF in highly degenerated IVDs, due to the lack of structural integrity to physically maintain the cells after injection and an appropriate biological environment important to prevent cell apoptosis. Therefore, patients suffering moderate IVD degeneration may benefit most from this type of treatment [109,110,111]. Other restrictions relevant to the cell-based therapy is how to maintain cells at the injection site, preserve their ideal population, and determine the cell dose per injection. 

The prevention of cell apoptosis in the AF with avascular structure and poor nutrition is a notable challenge. Indeed, the IVD cellular metabolism is responsible for ECM homeostasis occurring via anaerobic glycolysis pathway with the conversion of glucose to lactic acid. To maintain cell activity and balance the catabolism-to-anabolism ratio, the supply of adequate glucose and appropriate removal of the lactic acid is crucial. Degenerated IVDs or ruptured AF suffer low swelling capacity, due to changes in the quantity and composition of the ECM, with weak nutrition–waste exchange leading to the accumulation of lactic acid. This results in a remarkable drop in pH across the AF that impairs cellular metabolism and increases the risk of cell apoptosis [112,113,114]. Low healing capacity is another restriction towards AF regeneration using cell-based therapies. During the healing process, annular lesions in the posterior AF have been observed to be filled initially with fibrin, blood, and cartilage debris and replaced with a thin layer of fibrous tissue over six months [115]. Studies have shown that almost more than one year is required for the production of collagen fibers with sufficient concentration to improve AF structural integrity [116,117]. Recent attempts to regenerate the AF have been more focused on the stimulation of the resident cells rather than increasing their population. One study suggested that increasing the rate of ECM synthesis was achievable by the transplantation of autologous stem cells to inhibit cell apoptosis [118]. 

Studies have shown that direct injection of cells has failed to regenerate the AF due to the cell leakage from the defect, making the preservation of the ideal cell population, important to remodel the ECM, almost impossible. Injectable cell delivery systems including biodegradable hydrogels, commercially available collagen II gels (Atelocollagen^®^), and hyaluronan gels have been employed to promote cell-based strategies via increasing cell retention time at the injection site to prevent leakage-induced-osteophyte formation [88,119,120,121,122,123,124,125]. Studies have revealed that cell-seeded hydrogels based on collagen type II significantly enhanced the rate of ECM synthesis, compared to those employed collagen type I [126,127]. Additionally, collagen gels seeded with ovine AF cells were used for the fabrication of heterogeneous constructs resembling the circumferential alignment of the AF [128]. The rate of degradation in biodegradable hydrogels was found to affect the rate of ECM biosynthesis, with the slower rates of degradation hindering ECM remodeling [129]. Employment of injectable cell-seeded hyaluronan gels was shown to promote ECM production, support the AF cells’ proliferation, and generate a highly conducive environment for chondrogenesis [130,131]. While the challenges of delivering high quality and viable cells at the point of care is a commercial and regulatory challenge, synthetic biomaterials might offer advantages. Despite being minimally invasive, injectable polymers have not been able to entirely address the current problems due to their low mechanical properties. The regenerative strategies to stop or reverse the degeneration process in IVDs are identified in Table 2.

## 5. Future Outlook

Understanding the mechanisms of IVD degeneration to stop or reverse this process or re-establishing function through therapies is a challenge at the intersection of biomaterials, biomechanics, clinical practice, and biology [132,133,134]. Despite intense research interest in this area, lack of suitable collaborations has been a severe limitation towards the creation of effective therapeutic devices to regenerate the AF. Yet those who are presently working at this multidisciplinary crossing point indicate the current challenge is to get these scientific cultures to work together. Already formed joint meetings to bridge these disparate disciplines is motivating; however, not adequate, evidenced by the unproductive outcome. Noticeably, it is important to develop a new standard for training the next generation of biomechanical and biomaterials engineers with a strong background in biochemistry, molecular, and cell biology. This new generation of engineers will be specifically able to offer a way of thinking that may contribute practically to unravel the inherent complexity of IVD and propose effective biologically-grounded engineering solutions to cure low back pain, regenerate IVDs, and design future medical devices.

Over decades, separate and independent approaches to find relative solutions for the NP replacement, AF repair, and IVD replacement have resulted in partial achievements. Attempts to replace the NP and keep the implant in place have failed in the absence of appropriate strategies for AF repair. However, there are newer designs of nucleus replacement undergoing clinical trials that are minimally invasive and use sophisticated delivery technology that may come to clinical use in the future [135]. Development of injectable biomaterials to simultaneously replace the NP and repair the AF is one step forward to resolving the problems associated with herniation [136]. Since the clinical translation of in vitro cell studies is yet challenging, the first but not the best approach will be the preparation of cell-free injectable biomaterials to effectively repair the AF defect in the short term [137]. The AF defects strongly change the mechanical properties of the IVD and activate catabolic routes that are responsible for accelerating IVD degeneration [138]. Indeed, the development of mechanically-reinforced injectable biomaterials that resemble tissue properties, although cell-free, provide opportunities to stop or decelerate the degeneration process. This approach may be an effective treatment for both young patients suffering herniation or those who are at the early stage of IVD degeneration. To achieve this, the development of new biomaterials and the employment of novel crosslinking agents is critical. One suggestion is the use of hyperbranched molecules in self-healing hydrogels to simultaneously increase the mechanical and swelling properties [139].

From a structural point of view, another key approach is a thorough understanding of the herniation pathway [18,140]. While a small number of microstructural and one biomechanical–microstructural studies have revealed the herniation pathway in the outer AF, more scientific explorations are required to address the herniation mechanism [124]. Since recent studies revealed that elastic fibers in the AF form an organized network across the AF, understanding of their role in the structural integrity of the healthy and degenerated IVDs is an important step towards developing new strategies to repair the AF. The design of new biomaterials mimicking the biochemical, mechanical, and morphological properties of elastic fibers may provoke the functionality of future repair strategies. One approach would be the formation of chemical bonding between injectable elastic-based biomaterials and the AF elastic network. This may result in more secure treatments reducing the risk of biomaterial leakage which is a common problem with existing injectable systems.

From a clinical point of view, more studies are required to address the impact of total discectomy on the structural integrity of AF tissue adjacent to the rapture site. Likely, one major reason for unsuccessful attempts to repair the AF after discectomy is harsh tissue removal during surgery. The understanding of the impact of discectomy after herniation on the structural integrity of AF remains an important mid- to long-term goal in spine research.

Future challenges toward effective AF repair strategies and IVD replacement include fabrication techniques that replicate the structural complexity of the tissue. Recent studies on presenting electrospun angle-ply structures and collagen gel composites were able to partially replicate the tissue structure [59,141,142,143]. However, the mechanical and morphological properties of the elastic fibers are missing pieces and their primary stability is an unsolved problem. Taking inspiration from the structural organization of elastic fibers and collagen bundles, 3D bioprinting may play an important role in the fabrication of IVD implants to restore the AF function and properties. The addition of calcified biomaterials during 3D bioprinting, an option to captivate the endplate structures and properties, is an approach to resolve the primary stability issue via bone growth. While 3D bioprinting provides unique opportunities for the fabrication of complex constructs by combining cells, fibers, and biomaterials, it has been unexplored for IVD repair strategies [144,145,146]. The challenge ahead is the lack of suitable structural models recapitulating the multiscale hierarchy of the IVD structure [147]. The major drawback is the absence of fibril form in the current 3D IVD approaches [147,148], since the fibril topography was shown to have an impact on cell adhesion and phenotype [149,150]. Technical difficulties in the formulation of bioinks to retain appropriate shape fidelity and adjust their shear-thinning behavior remain challenging. The development of novel hydrogels with optimal mechanical and biochemical properties and modulation of viscosity (blending with thickeners) to enhance shape fidelity will be essential for successful 3D bioprinting of IVD constructs. To the best of our knowledge, there is a lack of bioink specifically designed for IVD construction and it is not clear whether available bioinks for regeneration of cartilage [151], cardiovascular tissues [152], heart valves [153], or other tissues [154,155,156] are appropriate for IVD bioprinting. Another challenge is optimizing printing parameters including the rate of printing, characteristics of bioinks (temperature, concentration, surface charge, and water content), the geometry of the nozzle, post-extrusion shape and size stability of the 3D bioprinted constructs, and crosslinking intervals [148,157]. These parameters critically influence the quality of the final constructs as well as directly affect the viability of cells, if included to the bioink, by being exposed to relatively high pressure during fabrication. Of particular interest is the lack of evidence demonstrating interactions amongst the optimized structural parameters of IVD and cells survival within IVD.

## 6. Conclusions

Damage to the AF and disintegration of the IVD structure are likely to result in IVD herniation causing low back pain. The main challenge to repair the AF is sealing the site of rupture to achieve a functional implant that integrates the host with match mechanical properties compared to the natural tissue. Unfortunately, despite intense research, attempts to repair the AF have failed so far and no effective strategy has translated into a successful clinical outcome. Although knowledge around IVD regeneration has significantly increased and new methods have developed, numerous attempts to regenerate the AF have not concluded to satisfactory results due to the lack of a method to secure the cells at the defect site. It seems that therapeutic strategies should strongly focus on the prevention of AF damage or techniques for AF reconstruction to preserve the structural integrity of the AF in IVDs. This will result in the restoration of the mechanical properties of IVD and the control of catabolic routes that are responsible for accelerating IVD degeneration. To develop effective repair and regenerative strategies, including mechanically stable cell-seeded hydrogel, challenges ahead are likely to be a thorough understanding of herniation pathway, role, and structural organization of fibrous component of the IVD, as well as incorporating advanced additive manufacturing approach. It is also important to mention that surgical procedures to treat herniation should avoid further damage to the AF as much as possible.

## Figures and Tables

**Table 1:** The strategies to repair AF and their feasibility.

Repair Strategies		
Strategy	Current Status	Feasibility
**Early AF closure device (Xclose^®^, Inclose^®^)**	Not available anymore	Not effective to stop re-herniation and improve the rate or strength of the AF healing.
**Suturing techniques**	Rarely used	Are not reliable to sustain complex cyclic loading and not efficient yet.
**Thermal therapies** **(pulse radiofrequency and intra-discal electro-thermal therapies)**	In use	Are likely to be effective to seal the structural defects in the mildly ruptured AF. Sever AF defects are unlikely to be treated and their contribution to IVD degeneration in the long term is not known.
**Advanced AF closure devices** **(Barricaid^®^)**	Clinical trial	Being effective to reduce the rate of re-herniation and alleviate pain. The key limitation is being different from the native tissue in terms of structure and property, hence it may accelerate the degeneration process.
**Injectable bio-adhesives**	Laboratory trial	The appropriate properties (i.e., adhesion, mechanical strength) and long-term capacity to resist high stresses during daily activities have remained a major concern.
**Polymeric implants**	Laboratory trial	Not sufficient data available to evaluate their feasibility and no further clinical practice was reported.

**Table 2:** Current regenerative strategies to hinder or reverse the degeneration process in IVDs.

Regenerative Strategies		
Strategy	Current Status	Feasibility
**Gene therapies**	Laboratory trial	Being effective in accelerating the regeneration process, stimulation of ECM remodeling, and prevention of progression to AF injury. The frequent observation of AF cell apoptosis in degenerated IVDs is a barrier.
**Biomolecule therapies** **(growth factors)**	Laboratory trial	Being effective in cell differentiation, enhancing the healing process, and upregulating of healthy cell marker genes. The short lifetime of biomolecules is a limitation that restrains the efficacy of biomolecules to regenerate AF.
**Cell therapies**	preclinical studies	Being successful in ECM remodeling via direct transplantation of proliferated mesenchymal stem cells into the degenerated AF. Unlikely to regenerate highly degenerated IVDs due to the lack of (1) structural integrity to physically maintain the cells after injection, and (2) an appropriate biological environment important to prevent cell apoptosis.
**Injectable cell delivery gels**	Laboratory trial	Being effective to promote cell-based strategies via increasing cell retention time at the injection site. Injectable polymers have not been able to entirely address the current problems due to their low mechanical properties.

## Abbreviations

LBP	Low back pain
IVD	Intervertebral disc
AF	Annulus Fibrosus
NP	Nucleus Pulposus
ECM	Extracellular Matrix
ILM	Inter-lamellar matrix
GDF	Growth differentiation factors
YLD	Years lived with disability
